# Supplementary material for: Seminoma and Embryonal Carcinoma Footprints Identified by Analysis of Integrated Genome-Wide Epigenetic and Expression Profiles of Germ Cell Cancer Cell Lines
Source: PLoS One. 2014 Jun 2;9(6):e98330. doi: 10.1371/journal.pone.0098330 (PMC4041891; doi:10.1371/journal.pone.0098330)
Supplement: File S1 — ZIP file containing DMRforPairs output for significant regions. Please start from the html files. (ZIP) [file pone.0098330.s008.zip › figures/13261.pdf]

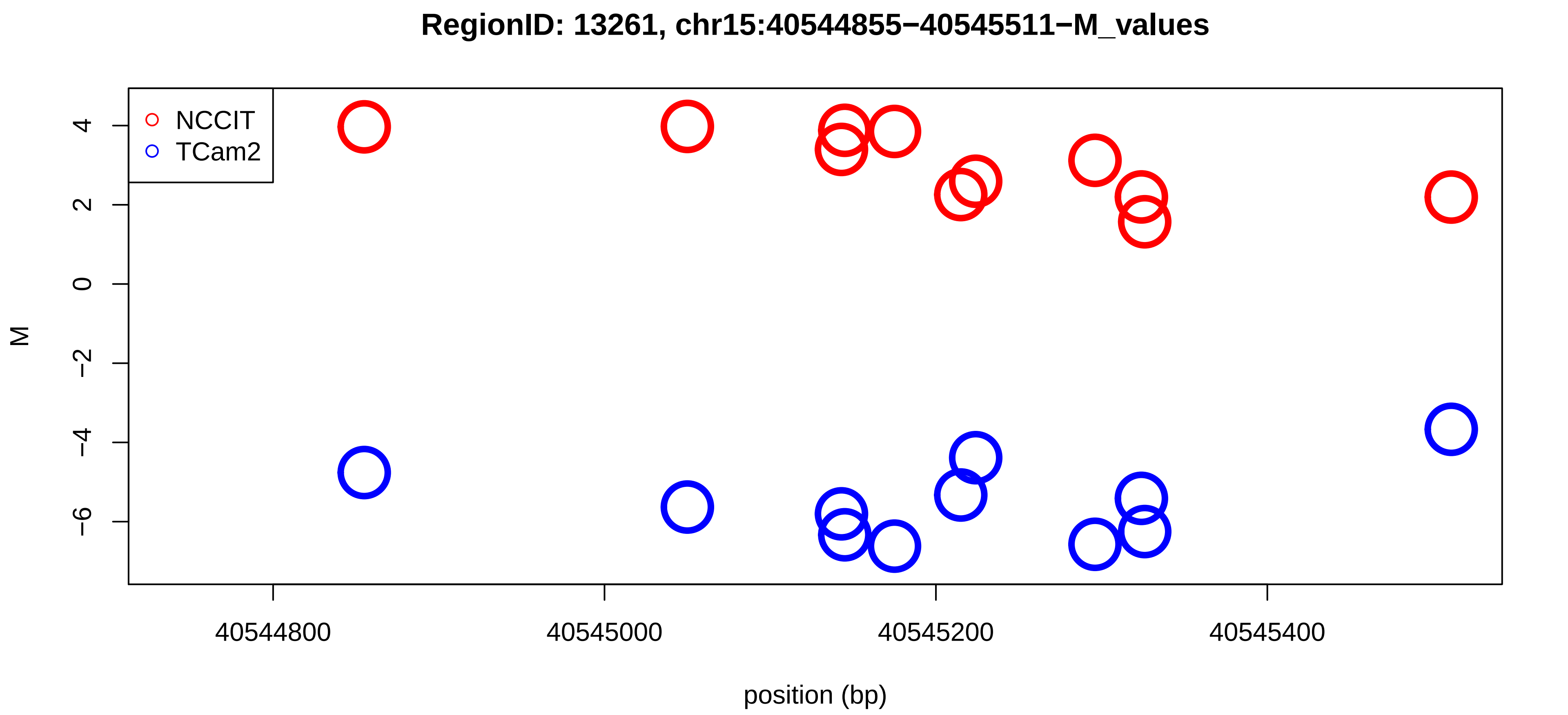

RegionID: 13261, chr15:40544855−40545511−M_values
NCCIT
TCam2
M
4
2
0
−2
−4
−6
40544800
40545000
40545200
40545400
position (bp)


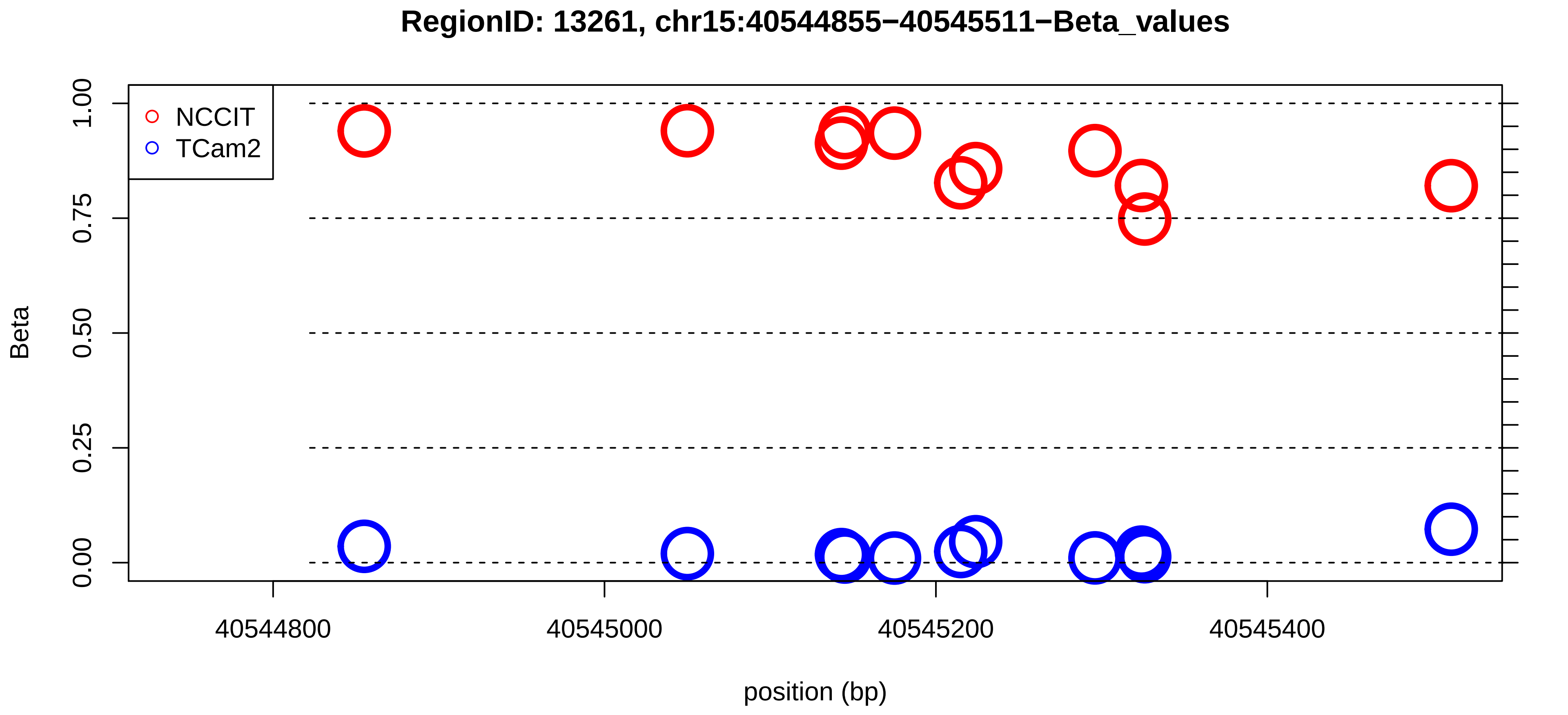

RegionID: 13261, chr15:40544855−40545511−Beta_values
NCCIT
TCam2
Beta
1.00
0.75
0.50
0.25
0.00
40544800
40545000
40545200
40545400
position (bp)

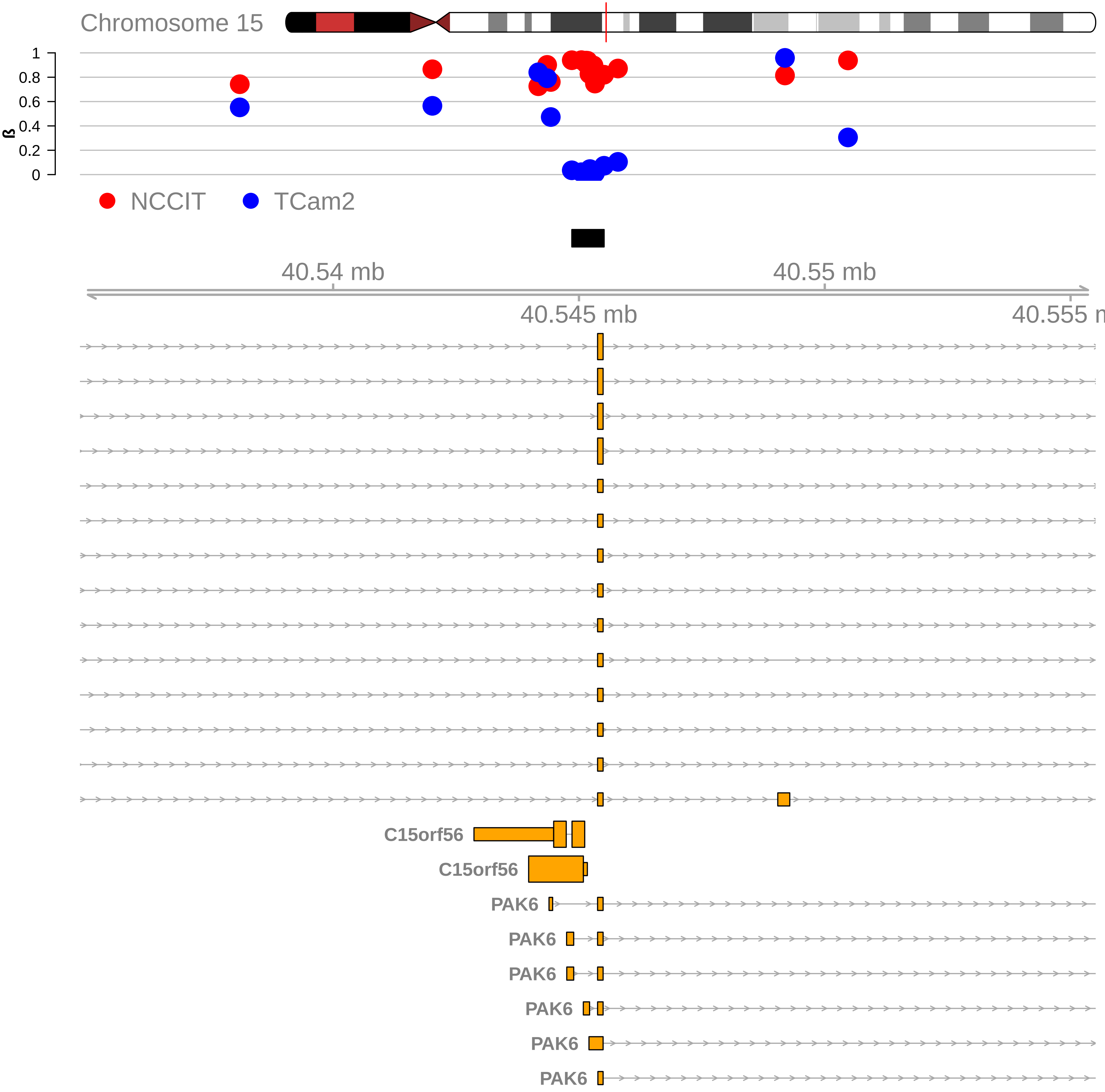

Chromosome 15
ß
1
0.8
0.6
0.4
0.2
0
NCCIT
TCam2
40.54 mb
40.55 mb
40.545 mb
40.555 mb
C15orf56
C15orf56
PAK6
PAK6
PAK6
PAK6
PAK6
PAK6
